# Burnout and coping among healthcare providers working in Saudi Arabia during the COVID-19 pandemic

**DOI:** 10.1186/s43045-021-00108-6

**Published:** 2021-06-10

**Authors:** Sumayah AlJhani, Hatim AlHarbi, Shahad AlJameli, Lama Hameed, Khozama AlAql, Mohammed Alsulaimi

**Affiliations:** 1 Department of Psychiatry, College of Medicine, Qassim University, Qassim, Saudi Arabia; 2 College of Medicine, Qassim University, Qassim, Saudi Arabia; 3 Department of Internal Medicine, Gastroenterology, King Saud Hospital, Qassim, Saudi Arabia

**Keywords:** Burnout, Copenhagen burnout inventory, Brief-COPE, Healthcare providers, COVID-19

## Abstract

**Background:**

Burnout is defined as a syndrome resulting from chronic workplace stress that has not been successfully managed. It is characterised by feelings of energy depletion or exhaustion, increased mental distance from one’s job and reduced professional efficacy. The COVID-19 pandemic has created unexpected demands on healthcare systems worldwide and they have experienced numerous stressors. As the coping is one of the stressors management strategies that may affect burnout, this is a descriptive cross-sectional study aimed to estimate the frequency and level of burnout and its association with coping strategies among physicians and nurses in Saudi Arabia during the COVID-19 Pandemic using Copenhagen Burnout Inventory and Brief-COPE.

**Results:**

Overall, 403 healthcare providers were recruited (85 physicians, 318 nurses). Personal, work-related and client-related burnout were detected among 67.5%, 68% and 58.3% of the respondents, respectively. The mean score for adaptive coping was (27.6 ± 10.3, median: 29 IQR: 14.0) out of 48, and the mean score for maladaptive coping was (14.2 ± 6.81, median: 14 IQR: 8.0) out of 36. Some factors associated with burnout were participants’ age group, professional position, number of family members and years of experience in the medical field. The personal, work-related and client-related burnout had inverse correlations with the overall adaptive coping category.

**Conclusion:**

The frequency of burnout during the COVID-19 pandemic, particularly among nurses, was significant. Burnout was also frequent among both the younger age group and those with fewer years of experience. Some predictors were identified as having a close person infected with COVID-19, being assigned to treat COVID-19 patients, longer working hours, having sleeping hours affected by the pandemic and experiencing verbal or physical abuse from patients. In addition to a significant correlation between the adaptive coping category and the three burnout dimensions.

## Background

Health is a human right, and it has a strong association with human development indicators [1]. One of the most important social determinants of health is exposure to stressful and harmful living and working conditions. Due to the nature of their work, healthcare workers (HCWs), especially physicians and nurses, can experience stressful and harmful working conditions and consequently risk developing negative health issues such as burnout [2]. Burnout is one of the primary factors affecting the quality of both their work and performance [1]. Burnout syndrome can affect individuals belonging to all professions and age ranges. Moreover, burnout is considered to have an important effect on the quality of life, and the risk of developing diseases [3], in addition to various psychological consequences [4].

The coronavirus disease 2019 (COVID-19) pandemic has spread rapidly and presented global health systems with serious challenges regarding identifying, managing and preventing infections and creating effective strategies to protect the public [5]. During the COVID-19 pandemic, frontline HCWs have experienced several psychosocial stressors and high volumes of work, which may affect their emotional and mental health and lead to burnout [6]. As this pandemic may foster distinctive workplace stress, recognising such stresses and identifying contributing factors and coping strategies to develop preventive measures is essential.

## Literature review

Burnout is defined by the International Classification of Diseases-11 as a ‘syndrome resulting from chronic workplace stress that has not been successfully managed’. It is characterised by feelings of energy depletion or exhaustion, increased mental distance from one’s job or feelings of negativism or cynicism related to one’s job, and reduced professional efficacy [7].

As a result of the unexpected demands on healthcare systems worldwide due to COVID-19, frontline HCWs have experienced numerous stressors, high work volumes, and sleep deprivation, which can increase the risk of burnout [8]. Increased work demands, worries about becoming infected with COVID-19 and social distancing may affect burnout [8]. Additionally, treating patients with COVID-19 can cause distress, depression, insomnia and anxiety [9].

Burnout may be accompanied by both physical and psychological symptoms including tiredness, disturbed sleep and appetite, physical pain, pessimism, indifference, irascibility and hesitation [10]. Ultimately, it affects quality of life and increases the risk of secondary psychiatric and physical illnesses [4].

HCWs, especially physicians and nurses, can be at high risk of burnout because of the nature of their work. The effects of burnout can negatively impact client care, consistency of care, and the risk of medical errors [1, 10]. Acknowledging the risk of burnout among HCWs may help decrease the stigma towards mental health concerns, and, in turn, help prevent burnout [5].

A global meta-analysis review reported that 11.23% of nurses have experienced burnout [11]. Furthermore, a systematic review concerning burnout among doctors revealed high levels of emotional exhaustion and depersonalisation and low levels of personal accomplishment [12].

HCWs exhibit higher levels of psychological distress and acute or post-traumatic stress while working with patients during outbreaks of viral infectious diseases when compared to controls who have lower levels of contact with such patients [5]. A study on HCWs who cared for patients with COVID-19 found that over half of the sample had high levels of burnout [13]. Similarly, another study found that, during pandemics, the average level of burnout among medical residents was higher than that of non-pandemic periods [14].

A study of frontline HCWs in Italy during the COVID-19 pandemic found that they had higher levels of emotional exhaustion than non-pandemic periods; however, relatively few HCWs had low levels of personal gratification and depersonalisation [6].

Physicians (especially residents) and nurses (particularly those working in acute critical care departments and those who have a history of depressive or anxiety disorders) are at high risk of burnout during the COVID-19 pandemic [13]. Common concerns for HCWs during the pandemic include infecting family members and insufficient access to protective equipment [15].

Coping styles are also associated with burnout. Lazarus and Folkman define coping as a set of cognitive and behavioural efforts that are applied to address the occurrence of internal or external demands considered to exceed one’s personal resources [16]. In the workplace, positive coping creates positive feelings that foster improved communication and occupational growth [3]. Furthermore, positive coping can inhibit the emergence of harmful health conditions, and manifest as problem-solving behaviour and positive appraisals, while negative coping can manifest as a distorted mindset [3]. Coping strategies used by HCWs, such as talking to senior staff and having hobbies, decreases the risk of burnout, while venting emotions and using substances increases the risk [17]. Furthermore, using emotions focused on coping rather than the problem increase the risk of burnout [18]. Overall, positive coping has a positive impact on HCWs’ psychological state, while negative coping is related to burnout [3, 19].

While numerous studies have discussed the psychological impact and prevalence of burnout syndrome among HCWs in developed and westernised countries during the COVID-19 pandemic, few studies have been conducted in Middle Eastern countries, including Saudi Arabia. Notably, a systematic review published in 2019 (prior to the COVID-19 pandemic) reported the presence of high rates of burnout syndrome among HCWs in Middle Eastern countries [20]. Thus, the mental and physical impact of COVID-19 among HCWs necessitates further consideration. Consequently, the present study aimed to estimate the frequency and level of burnout among physicians and nurses working in Saudi Arabia during the COVID-19 pandemic, determine the contributing factors and identify the coping strategies that such workers use.

## Methods

### Study design and study area

This study featured a descriptive cross-sectional design, and all regions of Saudi Arabia were targeted for the sample recruitment. All physicians and nurses registered in the Saudi Commission for health specialities during COVID-19 were included. The sample was calculated as having a 95% confidence interval and margin of error of ± 5%, and the required sample was 384.

On 2 March, 2020, the Saudi Ministry of Health announced the first case of COVID-19 in the country [21]. Response collection started in July and continued over 2 months.

### Research instrument and validation

An online survey was distributed through the Saudi Commission for Health Specialties and social media targeting physicians and nurses of all specialties and levels. The survey comprised three components: demographic data, the Copenhagen Burnout Inventory (CBI) and the Brief-COPE (Coping Orientation to Problems Experienced). The latter two questionnaires have both been used in previous studies of HCWs [22, 23]; an English version of both questionnaires was used.

### Copenhagen burnout inventory and brief-COPE

The CBI is a self-administered questionnaire developed by Kristensen et al. [24] which focuses on three dimensions: personal burnout, work-related burnout and client-related burnout. It contains 19 items, and responses to these items are rated on a five-point scale (comprising ‘always’, ‘often’, ‘sometimes’, ‘seldom’ and ‘never/almost never’, respectively; or ‘to a very high degree’, ‘to a high degree’, ‘somewhat’, ‘to a low degree’ and ‘to a very low degree’, respectively). It has satisfactory reliability and validity [24]. Average scores are calculated for each domain, with an average score of 50% or above indicating burnout [25]. Regarding overall reliability, CBI Cronbach’s alpha was 0.953 (95.3%) for the CBI, indicating excellent internal consistency.

The Brief COPE comprises 14 subscales; each of which is measured using two items. Responses are given using a four-point Likert scale ranging from 0 to 3. These subscales can be categorised into approach/avoidant or adaptive/maladaptive forms of coping behaviours, respectively. The adaptive coping category contains 16 items (giving a possible range of 0–48), and includes the active coping, planning, positive reframing, acceptance, humour, religion, using emotional support and instrumental support subscales. Meanwhile, the maladaptive coping category contains 12 items (giving a possible range of 0–36) and includes the self-distraction, denial, venting, substance use, behavioural disengagement and self-blame subscales [26–28]. Regarding overall reliability for the present study, Cronbach’s alpha was 0.889 (88.9%), indicating very good internal consistency.

### Statistical analyses

Descriptive statistics were presented in the form of counts, proportions (%), medians (min–max), means and standard deviations (SDs) where appropriate. The scores for the CBI dimensions were compared to the respondents’ sociodemographic characteristics using the Mann-Whitney *U* test and Kruskal-Wallis test. *P* < 0.05 was considered to indicate statistical significance. Additionally, using the Kolmogorov-Smirnov and Shapiro-Wilk tests, normality, statistical interactions and collinearity (i.e. variance inflation factor) were assessed. The data followed an abnormal distribution; thus, non-parametric tests were applied. Correlation was conducted to determine the linear relationship between the Brief-COPE domains and the CBI dimensions. Data analyses were performed using SPSS Version 21 (Armonk, New York, IBM Corporation).

### Ethical considerations

All subjects gave their informed written online consent for inclusion before participation. The study protocol was approved by Qassim Regional Research Ethical Committee.

## Results

We analysed 403 HCWs (318 nurses and 85 physicians) and measured their burnout status during the COVID-19 pandemic. The most common age group was 22–35 years (60.5%), and most participants were female (75.9%). Over half (54.8%) were non-Saudis, and almost 60% were married. Furthermore, the majority of respondents were living in the central region (52.4%), and most lived with 1–5 family members. Of the 85 physicians, 35.3% were resident physicians and 32.9% were consultants. Of the 318 nurses, most had a bachelor’s degree (71.7%).Over half of the respondents reported having more than six years of experience in the medical field. Almost half (46.9%) indicated that they usually earned SAR 4000–8000 per month. When asked if their income had been affected by the pandemic, 60% indicated that it had not been affected, 20.3% said that it had increased and 19.6% said that it had decreased. Additionally, approximately 68% were working in government hospitals. Finally, 10.9% of the respondents reported having chronic diseases.

**Fig. 1:**
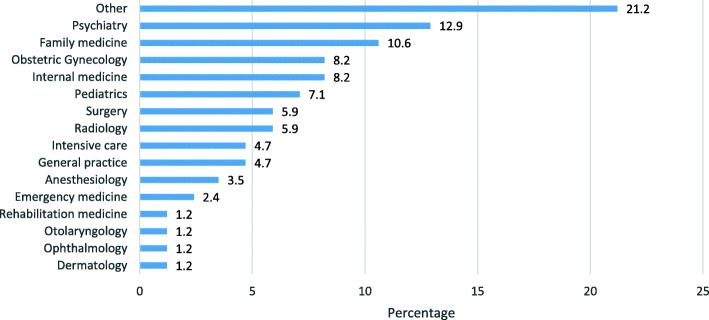
Physicians’ specialties

Among physicians, the most common specialties were psychiatry (12.9%) and family medicine (10.6%) (Fig. 1).

**Fig. 2:**
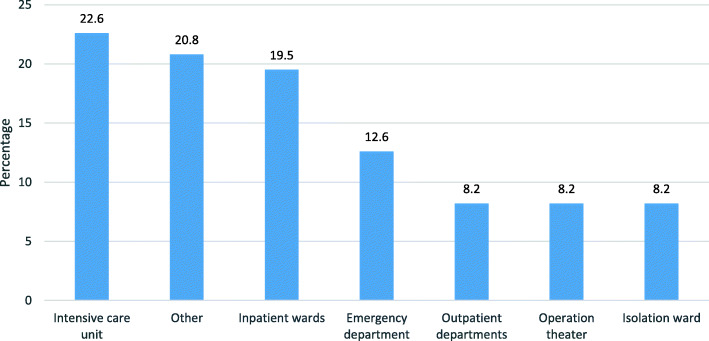
Nurses’ departments

Most nurses worked at the intensive care unit (22.6%) followed by the inpatient ward (19.5%) and emergency room (12.6%) (Fig. 2).

Based on descriptive statistics of the CBI dimensions and brief cope, it was found that burnout median score was higher on personal related burnout (mean 58.9 ± 26.5, median 58.3, IQR 37.5) followed by work related burnout dimension (mean 56.9 ± 22.6, median 57.1, IQR 28.6), while the least was client-related burnout (mean 51.5 ± 26.1, median 50, IQR 37.5). Concerning brief cope scales which were subdivided into two subscales (adaptive and maladaptive coping subscales). The overall median adaptive coping subscale score was 29 (IQR 14.0, mean 27.6 ± 10.3). Among its domains, religion (median 4.0, IQR 3, mean 4.03 ± 1.82), acceptance (median 4.0, IQR 2, mean 3.81 ± 1.73), active coping (median 4.0, IQR 2, mean 3.63 ± 1.61), planning (median 4.0, IQR 3, mean 3.57 ± 1.67) and positive reframing (median 4.0, IQR 6, mean 3.71 ± 1.71) were higher. On the other hand, the overall median maladaptive coping subscale score was 14 (IQR 8.0, mean 14.2 ± 6.81). With regards to its domains, self-distraction (median 3.0, IQR 2, mean 3.29 ± 1.59), venting (median 3.0, IQR 2, mean 2.78 ± 1.60) and denial (median 3.0, IQR 3, mean 2.32 ± 1.79) had the highest median score (Table 1).

**Table 1:** Descriptive statistics of CBI and Brief COPE (*n* = 403)

CBI dimensions	Median	IQR
Personal burnout	58.3	37.5
Work-related burnout	57.1	28.6
Client-related burnout	50	37.5
Brief COPE		
**Adaptive coping subscales**	**29.0**	**14.00**
• Instrumental support	3.00	2.00
• Emotional support	3.00	2.00
• Active coping	4.00	2.00
• Planning	4.00	3.00
• Positive reframing	4.00	6.00
• Acceptance	4.00	2.00
• Humour	2.00	3.00
• Religion	4.00	3.00
**Maladaptive coping subscales**	**14.0**	**8.00**
• Self-distraction	3.00	2.00
• Denial	3.00	3.00
• Self-blaming	2.00	4.00
• Behavioural disengagement	2.00	3.00
• Venting	3.00	2.00
• Substance use	0.00	2.00

*CBI* Copenhagen Burnout Inventory

**Fig. 3:**
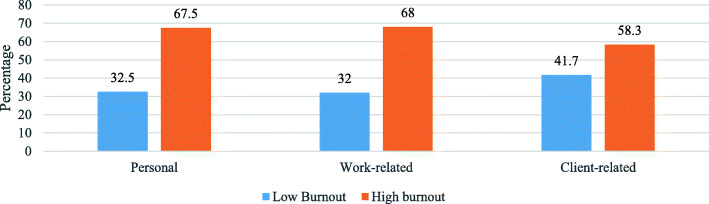
Level of burnout according to the three CBI dimensions

Figure 3 depicts the level of burnout among respondents in terms of each of the CBI’s dimensions. High personal, work-related and client-related burnout was detected among 67.5%, 68% and 58.3% of respondents, respectively, while low personal, work-related and client-related burnout was observed among 32.5%, 32% and 41.7% of respondents, respectively.

We performed a correlation procedure to determine the linear agreement between the CBI dimensions and Brief-COPE domains (Table 2). The personal burnout dimension had inverse correlations with the overall adaptive coping category (*r* = − 0.116; *p* = 0.020) and the instrumental support (*r* = − 0.116; *p* = 0.020), emotional support (*r* = − 0.099; *p* = 0.047) and positive reframing (*r* = − 0.100; *p* = 0.045) subscales, respectively. Work-related burnout had negative correlations with the overall adaptive coping category (*r* = − 0.113; *p* = 0.023) and the emotional support (*r* = − 0.109; *p* = 0.029), active coping (*r* = − 0.196; *p* < 0.001), self-distraction (*r* = − 0.110; *p* = 0.027) and positive reframing (*r* = − 0.099; *p* = 0.048) subscales, respectively. Moreover, client-related burnout showed significant inverse correlations with the overall adaptive coping category (*r* = − 0.161; *p* = 0.001) and the active coping (*r* = − 240; *p* < 0.001), acceptance (*r* = − 0.127; *p* = 0.010), self-distraction (*r* = − 0.126; *p* = 0.010), positive reframing (*r* = − 0.176; *p* < 0.001) and religion (*r* = − 0.166; *p* = 0.001) subscales, respectively. Conversely, self-blaming had positive and significant correlations with both work-related (*r* = 0.136; *p* = 0.006) and client-related (*r* = 0.175; *p* < 0.001) burnout.

**Table 2:** Correlations (Pearson’s *r*) between the CBI dimensions and Brief-COPE domains (*n* = 403)

Brief-COPE domains	Burnout		
	Personal	Work-related	Client-related
Adaptive coping subscales	− 0.116*	− 0.113*	− 0.161**
Instrumental support	− 0.116*	− 0.092	− 0.092
Emotional support	− 0.099*	− 0.109*	− 0.071
Active coping	0.144**	− 0.196**	− 0.240**
Planning	− 0.051	− 0.062	− 0.062
Positive reframing	− 0.100*	− 0.099*	− 0.176**
Acceptance	− 0.057	− 0.072	− 0.127*
Humour	− 0.089	− 0.043	− 0.037
Religion	− 0.040	− 0.052	− 0.166**
Maladaptive coping subscales	− 0.036	− 0.007	0.017
Self-distraction	− 0.084	− 0.110*	− 0.126*
Denial	− 0.057	− 0.048	− 0.004
Self-blaming	0.070	0.136**	0.175**
Behavioural disengagement	0.064	0.058	0.060
Venting	− 0.047	− 0.033	− 0.043
Substance use	− 0.085	− 0.038	− 0.010

* Correlation is significant at the 0.05 level (two-tailed); **correlation is significant at the 0.01 level (two-tailed)

When measuring the association between the CBI dimensions and respondents’ sociodemographic characteristics, respondents in the younger age group (22–35 years) showed significantly higher burnout in the personal (*T* = 2.388; *p* = 0.025), work-related (*T* = 3.102; *p* = 0.001) and client-related (*T* = 3.192; *p* = 0.002) dimensions when compared to those aged over 35 years. When compared to physicians, nurses showed significantly higher burnout in the personal (*T* = − 3.292; *p* = 0.002), work-related (*T* = − 4.594; *p* < 0.001) and client-related (*T* = − 3.024; *p* = 0.001) dimensions. Similarly, respondents living with 6–10 family members exhibited significantly higher burnout in the work-related (*F* = 2.152; *p* = 0.031) and client-related (*F* = 2.659; *p* = 0.048) dimensions than those living with other numbers of family members. Furthermore, nurses with postgraduate education showed significantly higher burnout in the personal (*F* = 2.805; *p* = 0.020) and work-related (*F* = 3.158; *p* = 0.009) dimensions when compared to other nurses. Those with experience of 6 years or less in the medical field exhibited significantly higher burnout scores in the personal (*T* = 2.377; *p* = 0.008), work-related (*T* = 3.107; *p* = 0.001) and client-related (*T* = 2.975; *p* = 0.003) dimensions when compared with those with more experience. Respondents with less income (≤ 8000 SAR) showed significantly higher burnout scores for the personal (*F* = 5.365; *p* = 0.008) and work-related (*F* = 4.678; *p* = 0.019) burnout dimensions when compared to those with higher incomes. Those who reported an increased income during the pandemic showed significantly higher burnout scores in the personal burnout dimension (*F* = 5.718; *p* = 0.002) when compared to those whose income had decreased. However, those whose income had decreased exhibited higher burnout scores in the work-related dimension (*F* = 3.149; *p* = 0.017) when compared to those whose income had increased. Finally, respondents working at government hospitals had significantly higher burnout scores for the personal dimension (*F* = 3.171; *p* = 0.021) when compared to those working at other hospitals (Table 3).

**Table 3:** Respondents’ sociodemographic characteristics (*n* = 403) and statistical associations between the CBI subscales

Factor	Burnout			
	*N* (%)	Personal Mean ± SD Total score (100)	Work-related Mean ± SD Total score (100)	Client-related Mean ± SD Total score (100)
Age group^a^				
22–35 years	244 (60.5%)	61.4 ± 25.5	59.7 ± 23.4	54.8 ± 27.1
> 35 years	159 (39.5%)	55.0 ± 25.9	52.7 ± 20.7	46.5 ± 23.7
*T* test		*2.388*	*3.102*	*3.192*
*P* value		0.025**	0.001**	0.002**
Gender^a^				
Male	97 (24.1%)	58.4 ± 27.5	55.9 ± 23.4	55.0 ± 23.4
Female	306 (75.9%)	59.0 ± 26.2	57.3 ± 22.4	50.4 ± 26.8
*T* test		− *0.215*	− *0.550*	*1.513*
*P* value		*0.920*	*0.779*	*0.131*
Nationality^a^				
Saudi	182 (45.2%)	59.4 ± 26.3	57.8 ± 22.6	53.7 ± 25.4
Non-Saudi	221 (54.8%)	58.4 ± 26.7	56.2 ± 22.7	49.8 ± 26.5
*T* test		*0.379*	*0.703*	*1.485*
*P* value		*0.694*	*0.222*	*0.076*
Marital status^a^				
Unmarried	173 (42.9%)	60.9 ± 26.1	58.2 ± 23.1	52.8 ± 26.5
Married	230 (57.1%)	57.3 ± 26.7	56.1 ± 22.3	50.6 ± 25.8
*T* test		*1.384*	*0.922*	*0.837*
*P* value		*0.179*	*0.313*	*0.234*
Position^a^				
Physician	85 (21.1%)	50.6 ± 28.5	47.2 ± 24.1	44.0 ± 25.1
Nurse	318 (78.9%)	61.1 ± 25.5	59.6 ± 21.5	53.6 ± 25.9
*T* test		− *3.292*	− *4.594*	− *3.024*
*P* value		0.002**	<0.001**	0.001**
Working region^a^				
Central region	211 (52.4%)	58.2 ± 24.4	56.7 ± 21.8	52.2 ± 25.6
Northern region	37 (09.2%)	56.1 ± 32.1	49.4 ± 22.4	43.5 ± 29.0
Eastern region	58 (14.4%)	61.2 ± 26.1	61.6 ± 20.2	53.4 ± 25.5
Western region	55 (13.6%)	58.9 ± 29.8	57.3 ± 26.3	50.9 ± 24.8
Southern region	42 (10.4%)	61.7 ± 27.6	57.9 ± 23.9	53.5 ± 27.9
*F* test		*0.371*	*1.688*	*1.062*
*P* value		*0.881*	*0.166*	*0.238*
Number of family members^b^				
None	46 (11.4%)	54.4 ± 28.0	51.8 ± 21.4	43.7 ± 26.1
1–5	262 (65.0%)	58.6 ± 25.8	56.5 ± 22.6	51.7 ± 26.4
6–10	71 (17.6%)	64.4 ± 24.4	62.2 ± 22.4	57.2 ± 24.1
> 10	24 (06.0%)	54.3 ± 34.8	55.8 ± 24.0	48.6 ± 25.8
*F* test		*1.709*	*2.152*	*2.659*
*P* value		*0.135*	0.031**	0.048**
Physicians’ professional rank (*n* = 85)^a^				
Service	06 (07.1%)	38.9 ± 20.0	39.9 ± 21.4	37.5 ± 20.6
Resident trainee	30 (35.3%)	58.9 ± 30.5	49.8 ± 26.3	42.6 ± 26.7
Consultant	28 (32.9%)	46.1 ± 28.4	48.1 ± 23.0	45.9 ± 22.2
Specialist	21 (24.7%)	48.0 ± 26.5	44.4 ± 24.1	45.2 ± 29.9
*F* test		*1.497*	*0.396*	*0.232*
*P* value		*0.271*	*0.861*	*0.756*
Nurses’ education level^b^				
Diploma	39 (12.3%)	52.6 ± 26.4	51.6 ± 15.2	50.7 ± 22.0
Bachelor’s	228 (71.7%)	61.8 ± 25.1	60.5 ± 22.5	53.2 ± 27.2
Masters or PhD	51 (16.1%)	64.7 ± 25.5	61.3 ± 20.2	57.3 ± 23.2
*F* test		*2.805*	*3.158*	*0.769*
*P* value		0.020**	0.009**	*0.489*
Years of experience in the medical field^a^				
≤ 6 years	181 (45%)	62.8 ± 27.0	60.8 ± 24.1	55.8 ± 27.2
> 6 years	222 (55.1%)	55.6 ± 25.6	53.8 ± 20.9	48.1 ± 24.7
*T* test		*2.737*	*3.107*	*2.975*
*P* value		0.008**	0.001**	0.003**
Monthly income (SAR)^b^				
≤ 8000	203 ((50.4%)	61.6 ± 25.8	59.2 ± 22.2	52.8 ± 26.4
8001–15,000	101 (25.1%)	55.3 ± 25.9	52.0 ± 23.6	50.1 ± 26.4
> 15,000	99 (24.6%)	48.3 ± 26.9	50.1 ± 23.0	44.7 ± 22.8
*F* test		*5.365*	*4.678*	*1.765*
*P* value		0.008**	0.019**	*0.188*
Income affected by COVID-19^b^				
Yes, higher	82 (20.3%)	63.3 ± 28.6	58.4 ± 25.6	53.5 ± 25.1
Yes, lower	79 (19.6%)	65.2 ± 26.1	61.9 ± 20.9	55.3 ± 23.6
No change	242 (60.0%)	55.3 ± 25.3	54.8 ± 21.9	49.7 ± 27.1
*F* test		*5.718*	*3.149*	*1.658*
*P* value		0.002**	0.017**	*0.154*
Working sector^b^				
Primary healthcare	71 (17.6%)	56.5 ± 23.5	52.9 ± 20.4	49.0 ± 21.2
Government hospital	272 (67.5%)	61.4 ± 26.2	59.1 ± 23.3	52.7 ± 27.3
Private hospital	39 (09.7%)	52.2 ± 29.2	53.9 ± 18.0	52.2 ± 23.3
University hospital	21 (05.2%)	47.4 ± 29.5	47.9 ± 25.2	44.2 ± 29.4
*F* test		*3.171*	*2.980*	*0.950*
*P* value		0.021**	*0.063*	*0.533*
Has a chronic disease^a^				
Yes	44 (10.9%)	58.2 ± 26.0	54.9 ± 22.3	52.1 ± 22.9
No	359 (89.1%)	58.9 ± 26.5	57.2 ± 22.7	51.5 ± 26.5
*T* test		− *0.170*	− *0.648*	*0.146*
*P* value		*0.975*	*0.601*	*0.996*

**significant at the *p* < 0.05 level

^a^*p* value has been calculated using the Mann Whitney *U* test

^b^*p* value has been calculated using the Kruskal-Wallis test

Table 4 presents data concerning respondents’ experiences during the COVID-19 pandemic, as well as other related variables. Most of the participants did not have previous experience with an infectious epidemic/pandemic (71%) or infection control (54.1%). Only 14.4% became infected with COVID-19, while 65.3% were tested, 52.6% were quarantined, and for more than half, a close person (e.g. family member, friend or co-worker) had been infected. Further, 56.3% of participants were assigned to treat patients with COVID-19 while 41.4% were assigned to a different specialty during COVID-19. About two-third of the participants’ working hours were affected during the pandemic: for 44.9%, working hours increased to more than 12 h while more than half of their sleeping hours were affected. Before the pandemic, 81.1% were getting at least 6–9 h of sleep; during the pandemic, 53.3% were getting less than 6 h of sleep. Only 18.9% received incentives during the pandemic at the time of the study and 33.7% were exposed to verbal or physical violence.

**Table 4:** Respondents’ experiences during the COVID-19 pandemic (*n* = 403) and statistical associations between the CBI subscales

Factor	Burnout			
	*N* (%)	Personal Mean ± SD Total score (100)	Work-related Mean ± SD Total score (100)	Client related Mean ± SD Total score (100)
Previous experience of an infectious epidemic/pandemic				
Yes	117 (29.0%)	57.8 ± 29.5	57.5 ± 24.7	52.4 ± 28.4
No	286 (71.0%)	59.3 ± 25.1	56.7 ± 21.8	51.2 ± 25.1
*T-test*		− *0.507*	*0.313*	*0.398*
*P-value*		*0.977*	*0.686*	*0.740*
Previous experience of infection control				
Yes	185 (45.9%)	58.7 ± 27.6	58.6 ± 23.4	53.4 ± 26.8
No	218 (54.1%)	59.0 ± 25.5	55.6 ± 21.9	49.9 ± 25.4
*T* test		− *0.115*	*1.305*	*1.353*
*P* value		*0.960*	*0.163*	*0.159*
Infected with COVID-19				
Yes	58 (14.4%)	61.9 ± 26.8	60.7 ± 22.1	55.5 ± 25.8
No	345 (85.6%)	58.4 ± 26.4	56.3 ± 22.7	50.9 ± 26.1
*T* test		*0.969*	*1.346*	*1.238*
*P* value		*0.305*	*0.216*	*0.210*
Tested for COVID-19				
Yes	263 (65.3%)	59.6 ± 26.4	57.9 ± 22.9	52.3 ± 25.5
No	140 (34.7%)	57.6 ± 26.5	55.1 ± 21.9	50.2 ± 27.2
*T* test		*0.698*	*1.185*	*0.765*
*P* value		*0.563*	*0.386*	*0.523*
Quarantined as a result of COVID-19				
Yes	191 (47.4%)	61.9 ± 25.9	58.3 ± 22.6	51.9 ± 25.6
No	212 (52.6%)	56.2 ± 26.7	55.7 ± 22.6	51.2 ± 26.6
*T* test		*2.192*	*1.134*	*0.277*
*P* value		0.024**	*0.301*	*0.956*
Close person (e.g. family member, friend or co-worker) infected with COVID-19				
Yes	231 (57.3%)	63.4 ± 25.1	60.9 ± 21.7	54.9 ± 24.1
No	172 (42.7%)	52.8 ± 27.1	51.6 ± 22.8	46.9 ± 27.9
*T* test		*4.033*	*4.203*	*3.117*
*P* value		< *0.001* **	< *0.001* **	*0.008* **
Assigned to work in a different specialty due to COVID-19				
Yes	167 (41.4%)	65.6 ± 25.3	61.2 ± 22.9	56.3 ± 25.7
No	236 (58.6%)	54.1 ± 26.3	53.9 ± 21.9	48.2 ± 25.9
*T* test		*4.380*	*3.242*	*3.124*
*P* value		< 0.001**	0.001**	0.003**
Assigned to treat patients with COVID-19				
Yes	227 (56.3%)	63.9 ± 25.5	62.4 ± 22.8	56.7 ± 25.9
No	176 (43.7%)	52.3 ± 24.9	49.9 ± 20.4	44.8 ± 24.7
*T* test		*4.469*	*5.712*	*4.659*
*P* value		< 0.001**	< 0.001**	< 0.001**
Received incentives from work during COVID-19^a^				
Yes	76 (18.9%)	61.2 ± 28.4	60.3 ± 24.4	57.5 ± 24.5
No	327 (81.1%)	58.3 ± 26.0	56.2 ± 22.1	50.2 ± 26.3
*T* test		*0.842*	*1.428*	*2.206*
*P* value		*0.313*	*0.120*	0.009**
Working hours affected by COVID-19^a^				
Yes	277 (68.7%)	61.7 ± 25.9	59.8 ± 22.4	53.7 ± 26.7
No	126 (31.3%)	52.7 ± 26.7	50.8 ± 21.9	46.8 ± 24.1
*T* test		*3.210*	*3.766*	*2.482*
*P* value		0.003**	< 0.001**	0.02**
Average daily working hours during COVID-19^b^				
8 h	96 (23.8%)	49.7 ± 25.0	47.5 ± 20.1	45.8 ± 24.6
> 8 h	75 (18.6%)	56.7 ± 23.9	56.1 ± 20.6	50.0 ± 23.7
> 10 h	51 (12.7%)	56.4 ± 27.4	50.2 ± 21.4	43.9 ± 26.8
> 12 h	181 (44.9%)	65.4 ± 26.4	64.2 ± 22.7	57.4 ± 26.4
*F* test		*8.313*	*14.637*	*6.339*
*P* value		< 0.001**	< 0.001**	0.001**
Sleeping hours affected by COVID-19^a^				
Yes	236 (58.6%)	65.2 ± 27.1	62.4 ± 22.9	56.5 ± 26.8
No	167 (41.4%)	50.0 ± 22.8	49.2 ± 19.8	44.5 ± 23.3
*T* test		*5.902*	*6.015*	*4.669*
*P* value		< 0.001**	< 0.001**	< 0.001**
Exposed to verbal or physical violence while working with COVID-19^a^ patients				
Yes	136 (33.7%)	67.5 ± 27.9	65.9 ± 24.4	62.7 ± 26.5
No	267 (66.3%)	54.5 ± 24.6	52.4 ± 20.3	45.9 ± 23.9
*T* test		*4.789*	*5.870*	*6.423*
*P* value		< 0.001**	< 0.001**	< 0.001**

**Significant at the *p* < 0.05 level

^a^*p* value calculated using the Mann-Whitney *U* test

^b^*p* value calculated using the Kruskal-Wallis test

When measuring the association between the burnout dimensions and the respondents’ characteristics, the respondents who had been quarantined as a result of COVID-19 showed significantly higher personal burnout scores (*T* = 2.192; *p* = 0.024) when compared to those who were not quarantined. Furthermore, those having close personal contact with COVID-19 patients showed significantly higher burnout scores in the personal (*T* = 4.033; *p* < 0.001), work-related (*T* = 4.203; *p* < 0.001) and client-related (*T* = 3.117; *p* = 0.008) dimensions when compared to other respondents. Those who had been assigned to work in different specialties as a result of the pandemic exhibited higher scores on the personal (*T* = 4.380; *p* < 0.001), work-related (*T* = 3.242; *p* = 0.001) and client-related (*T* = 3.124; *p* = 0.003) burnout dimensions than those who did not change specialty. Those who were assigned to treat patients with COVID-19 showed significantly higher burnout scores on personal (*T* = 4.469; *p* < 0.001), work-related (*T* = 5.712; *p* < 0.001) and client-related (*T* = 4.659; *p* < 0.001) dimensions than those who did not work with such patients, while those who received incentives from work during the pandemic showed significantly higher client-related burnout scores (*T* = 2.206; *p* = 0.009) than those who did not receive such incentives. Respondents whose working hours had been affected by the pandemic showed significantly higher personal (*T* = 3.210; *p* = 0.003), work-related (*T* = 3.766, *p* < 0.001) and client-related (*T* = 2.482; *p* = 0.029) burnout when compared to those whose working hours were unaffected. Respondents who worked an average of > 12 h a day during the pandemic exhibited significantly higher personal (*F* = 8.313; *p* < 0.001), work-related (*F* = 14.637; *p* < 0.001) and client-related (*F* = 6.339; *p* = 0.001) burnout scores than those who worked lesser hours, while those who stated that they slept for 10 h or more before the pandemic showed significantly higher personal burnout (*F* = 3.456; *p* = 0.038) than those who slept for fewer hours prior to the pandemic. Respondents whose sleeping hours were affected by the pandemic showed significantly higher personal (*T* = 5.902; *p* < 0.001), work-related (*T* = 6.015; *p* < 0.001) and client-related (*T* = 4.669; *p* < 0.001) burnout scores than those whose sleep was not affected. Incidentally, respondents who slept for an average of 10 h or more a night during the pandemic showed significantly higher personal burnout scores (*F* = 6.331; *p* = 0.001) than those who slept for less hours, but those who slept for less than 6 h were more strongly associated with work-related (*F* = 12.098; *p* < 0.001) and client-related (*F* = 7.501; *p* = 0.001) burnout when compared to the other respondents. Finally, respondents who had been exposed to verbal or physical violence from a patient confirmed as having or suspected of having COVID-19 during work showed significantly higher personal (*T* = 4.789; *p* < 0.001), work-related (*T* = 5.870; *p* < 0.001) and client-related (*T* = 6.423; *p* < 0.001) burnout scores when compared to those who had no such experiences (Table 4).

**Fig. 4:**
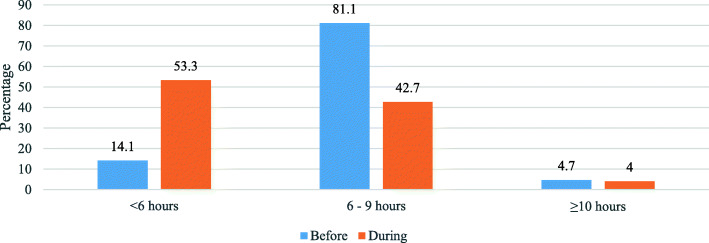
Comparison of regular sleeping hours before and during the COVID-19 pandemic

Figure 4 presents a comparison of the respondents’ regular sleeping hours before and during the pandemic.

## Discussion

The present study attempted to determine the frequency and level of burnout among physicians and nurses working in Saudi Arabia during the COVID-19 pandemic. Since there has been little research on this topic in Saudi Arabia, by clarifying the mental health status of HCWs during the pandemic, the present study may provide novel data. Such data are important because analysing HCWs’ psychological well-being can contribute to the development of effective coping strategies and other methods to help them address the adversities they have experienced as a result of the pandemic. Earlier studies on burnout have suggested possible causes of administration work, time pressure, independence with decisions and relations with patients and colleagues [29]. In the current study, significant burnout was detected among both physicians and nurses. Several previous studies have reported that, during the pandemic, burnout has been widely frequent among HCWs, which requires immediate attention [30–33]. Others, however, have reported moderate or minimal burnout [34–38]. In the present study, nurses were more strongly affected by burnout than physicians, similar to Matsuo et al.’s study [38], who measured burnout using the Maslach Burnout Inventory and found that its prevalence was higher among nurses than physicians. Similarly, Abdelhafiz et al. [36] reported that the prevalence of burnout is lower among doctors than other HCWs. Dinibutun [35] reported that physicians actively involved in treating patients with COVID-19 have lower burnout than physicians who are not actively involved with such patients.

We also found that those with fewer years of experience in the medical field were more likely to experience burnout when compared to those with longer tenure, similar to Matsuo et al.’s [38] results. Another study conducted in Saudi Arabia [34] also reported that HCWs in their initial two-year training period have a higher risk of experiencing burnout. Additionally, we found that younger HCWs (aged 22–35 years) were more likely to experience burnout than older HCWs (aged > 35 years). Conversely, Abdelhafiz et al. [36] indicated that older HCWs are more likely to develop burnout syndrome. In our study, across all three burnout dimensions, having a higher number of family members also predicts burnout, but gender and marital status produce no significant differences. However, Duarte et al. [32] and Khasne et al. [37] report that gender and marital status are both significantly associated with burnout. We also found that some workplace characteristics contribute to burnout among HCWs. For instance, having a close person contract COVID-19 was one of the factors associated with burnout, and this was also documented by Abdelhafiz et al. [36]. Likewise, our findings suggest that HCWs who are tasked with treating patients with COVID-19 and those who are assigned to work in different specialties because of COVID-19 are more likely to experience burnout, and similar findings have been presented in studies conducted in Singapore [31], Portugal [32] and the USA [33].

We found burnout to be frequent among those who had their working hours extended and those whose sleeping hours had decreased, as a result of the pandemic. Similarly, Matsuo et al. [38] suggest that decreased sleeping hours in conjunction with a desire for a reduced workload and a desire for appreciation or respect contributes to burnout among HCWs. Finally, we noted that experiencing verbal and physical abuse while working with COVID-19 patients is associated with burnout. Abdelhafiz et al. [36] also found that harassment by patients and one’s family members increases the risk of developing burnout syndrome.

We also analysed coping strategies used by HCWs to address the effects of burnout during the COVID-19 pandemic. The mean score for adaptive coping was 27.6 (SD: 10.3) out of 48 (57.5%), which can be categorised as a moderate use of adaptive coping strategies. However, the mean score for maladaptive coping was 14.2 (SD: 6.81) out of 36 (39.4%); these higher scores indicate poorer coping strategies. Both the adaptive and maladaptive coping means in this study were higher than previous studies [39]. Both adaptive and maladaptive coping had higher means among consultant physicians in Saudi Arabia during non-pandemic periods [27].

Regarding adaptive coping, the mean score for religion was the highest, followed by acceptance, and positive reframing, whereas for maladaptive coping, self-distraction had the highest mean and substance use had the lowest. Previous studies’ results on physicians and nurses in Saudi Arabia are consistent with the most and least frequent coping used; however, adaptive coping means were higher in comparison to our results [27, 40].

Further, we found an inverse significant correlation between the adaptive coping category and the three burnout dimensions, but the correlation between the maladaptive coping category and these dimensions was insignificant. The observed correlation between the adaptive coping category and burnout indicates that higher burnout scores lead to lower scores for adaptive coping. However, some of the adaptive coping domains also showed negative, significant correlations with the three burnout dimensions. For example, instrumental support was negatively correlated with personal burnout, while acceptance and religion were inversely correlated with work-related burnout. Emotional support was inversely correlated with both personal and work-related burnout, while positive reframing was negatively correlated with physical dimension but positively correlated to both the client and work-related dimension. For the maladaptive coping category, self-distraction showed an inverse correlation with both work-related and client-related burnout, but self-blaming was positively correlated with both work-related and client-related burnout.

Previous studies during COVID-19 have found positive reframing, humour and acceptance to be associated with better mental health, while self-distraction, self-blame and venting have been associated with poor mental health [41]. Others have found emotional and avoidance coping to be associated with stress, anxiety and depression [42] in addition to disengagement’s association with distress [43]. There has been a consistent correlation between burnout and dysfunctional coping among students [44] and educators [45]. Additionally, the use of adaptive coping reflects good psychological well-being, which leads to better and safer practice [46]. The current study is the first Saudi Arabia-based study to measure the linear agreement between burnout and coping strategies; the findings could serve as a basis for future reference. However, as this study was conducted during the COVID-19 pandemic, the questionnaire could only be distributed online; this may have affected the response rate and distribution of the sample.

## Conclusions

We found that the frequency of burnout among HCWs, particularly nurses, in Saudi Arabia during the COVID-19 pandemic is significant. Burnout is also frequent among the younger age groups and those in the early years of their medical careers. Furthermore, having a close person infected with COVID-19, being assigned to treat patients with COVID-19, having working hours affected by the pandemic, having longer daily working hours, having sleeping hours affected by the pandemic and experiencing verbal or physical abuse from patients with COVID-19 are also predictors of burnout among HCWs.

It is necessary to pay attention to the mental health status of these HCWs because they are on the frontline of combatting the current pandemic. Institutional management should take proactive steps to help HCWs with suspected burnout. Early intervention is important to improve positive well-being [47] and this could include the implementation of a recovery plan for such HCWs and the development of strategies for coping and self-care. Through such efforts, the psychological well-being of HCWs can be protected during the current crisis.

## Data Availability

Data available on request due to restrictions (e.g. privacy or ethical). The data presented in this study are available on request from the corresponding author. The data are not publicly available due to privacy agreement.

## Abbreviations

HCWs: Healthcare workers
COVID-19: Coronavirus disease 2019
CBI: Copenhagen Burnout Inventory
Brief-COPE: Coping Orientation to Problems Experienced
